# Contribution of *STAT4* gene single-nucleotide polymorphism to systemic lupus erythematosus in the Polish population

**DOI:** 10.1007/s11033-012-1752-3

**Published:** 2012-06-24

**Authors:** Piotr Piotrowski, Margarita Lianeri, Mariusz Wudarski, Marzena Olesińska, Paweł P. Jagodziński

**Affiliations:** 1 Department of Biochemistry and Molecular Biology, Poznań University of Medical Sciences, 6 Święcickiego St., 60-781 Poznan, Poland; 2 Institute of Rheumatology, Warsaw, Poland

**Keywords:** SLE, STAT4, Polymorphism

## Abstract

The *STAT4* has been found to be a susceptible gene in the development of systemic lupus erythematosus (SLE) in various populations. There are evident population differences in the context of clinical manifestations of SLE, therefore we investigated the prevalence of the *STAT4 G* > *C* (rs7582694) polymorphism in patients with SLE (n = 253) and controls (n = 521) in a sample of the Polish population. We found that patients with the *STAT4* C/G and CC genotypes exhibited a 1.583-fold increased risk of SLE incidence (95 % CI = 1.168–2.145, *p* = 0.003), with OR for the C/C versus C/G and G/G genotypes was 1.967 (95 % CI = 1.152–3.358, *p* = 0.0119). The OR for the *STAT4* C allele frequency showed a 1.539-fold increased risk of SLE (95 % CI = 1.209–1.959, *p* = 0.0004). We also observed an increased frequency of *STAT4* C/C and C/G genotypes in SLE patients with renal symptoms OR = 2.259 (1.365–3.738, *p* = 0.0014), (*p*_corr_ = 0.0238) and in SLE patients with neurologic manifestations OR = 2.867 (1.467–5.604, *p* = 0.0016), (*p*_corr_ = 0.0272). Moreover, we found a contribution of *STAT4* C/C and C/G genotypes to the presence of the anti-snRNP Ab OR = 3.237 (1.667–6.288, *p* = 0.0003), (*p*_corr_ = 0.0051) and the presence of the anti-Scl-70 Ab OR = 2.665 (1.380–5.147, *p* = 0.0028), (*p*_corr_ = 0.0476). Our studies confirmed an association of the *STAT4* C (rs7582694) variant with the development of SLE and occurrence of some clinical manifestations of the disease.

**Electronic supplementary material:**

The online version of this article (doi:10.1007/s11033-012-1752-3) contains supplementary material, which is available to authorized users.

## Introduction

Systemic lupus erythematosus (SLE) is a chronic autoimmune disorder characterized by the development of an immune response directed against any parts of the host body [1]. The course of SLE is unpredictable, with periods of remission and flare-ups [1]. Moreover, this autoimmune disorder is vastly heterogeneous, with various clinical manifestations including malar and discoid rash, photosensitivity, arthritis, serositis, as well as renal, neurologic, hematologic, immunologic and mucocutaneous manifestations, and biosynthesis of a broad array of autoantibodies [1]. The occurrence of SLE is nine times frequent in pre-menopausal women than in men [1].

It is accepted that environmental factors together with genetic components are involved in the abnormal immune responses and pathogenesis of SLE [2–6]. Flare-ups of SLE can be triggered by various environmental components, such as exposure to ultraviolet light, drugs, chemicals, and viral infections [6]. Candidate gene and genome wide association studies revealed numerous susceptibility genes of SLE, and the association of some of these genes have been confirmed among distinct populations [3].

The immune cells from patients with SLE display many abnormalities, including reduced T cell cytotoxicity, abnormal function of CD4^+^ T cells, abnormal activation of B cells, and alterations in cytokine biosynthesis [7–9]. The *STAT* (*signal transducer and activator of transcription*) *4* gene is expressed in T and B cells, monocytes, macrophages, natural killer cells, and dendritic cells [10]. STAT4 is a transcription factor and a member of the STAT family [10]. Its expression may support the differentiation of immune cells to inflammatory subsets, production of inflammatory cytokines and autoantibodies, prevention of apoptosis, and presentation of autoantigens, which may promote the development of autoimmune diseases [10].

Several genome-wide association studies have identified *STAT4* as an SLE susceptible gene in Caucasian and Asian populations [4, 5]. Recently, many studies have demonstrated the contribution of intronic single nucleotide polymorphisms (SNPs) of *STAT4* G > C (rs7582694) and G > T (rs7574865) to the incidence of SLE and its clinical manifestations [11–19]. Both of these polymorphisms display complete linkage disequilibrium (LD) in Asian and Caucasian populations presented in HapMap CHB data (http://hapmap.ncbi.nlm.nih.gov/).

We studied the *STAT4* G > C (rs7582694) polymorphism distribution in SLE patients in a sample from a Polish cohort. As SLE is a heterogeneous disorder, we also assessed the association of these polymorphisms with various clinical symptoms of SLE and the production of autoantibodies.

## Patients and methods

### Patients and controls

Data for two hundred and fifty-three women fulfilling the American College of Rheumatology Classification criteria for SLE [20, 21] were collected in a random manner for the study at the Institute of Rheumatology in Warsaw, Poland (Table 1). Controls included five hundred and twenty-one unrelated healthy volunteers and healthy women selected during medical examination at the Institute of Mother and Child, Warsaw. Women with SLE and controls were of Polish and Caucasian origin and of a similar age. The mean age of SLE patients at diagnosis was 34 ± 8 years, and of controls 33 ± 7 years. All participating subjects provided written consent. The study procedures were approved by the Local Ethical Committee of Poznań University of Medical Sciences.

**Table 1:** Distribution of the *STAT4* G > C (rs7582694) polymorphisms among SLE patients with different clinical manifestations

Characteristic	Genotype distribution			
	G/G	G/C	C/C	Odds ratio (95 % CI), *p* ^c^
	(131)^a^	(94)^a^	(28)^a^	
Malar rash	74	53	15	
Discoid rash	39	28	8	
Photosensitivity	58	48	14	
Oral or nasopharyngeal	62	44	13	
Arthritis	30	22	6	
Serositis	23	16	5	
Renal	51	59	13	2.259 (1.365–3.738, *p* = 0.0014)^b^
Neurologic	15	27	6	2.867 (1.467–5.604, *p* = 0.0016)^b^
Hematologic	43	30	9	
Immunologic	61	44	13	
ANA	131	94	28	

^a^Absolute number of positive patients for G/G, G/C, C/C genotypes, respectively. Comparison of genotypes ^b^(C/C or G/C vs G/G genotype) between patients with and patients without a particular manifestation was performed by ^c^χ^2^ test

### Genotyping

DNA was isolated from peripheral leucocytes using a standard salting out procedure. Identification of the *STAT4* C > G (rs7582694) polymorphic variant was performed by polymerase chain reaction-restriction fragment length polymorphism (PCR–RFLP). PCR was conducted employing primer pair 5′ ATCCAACTCTTCTCAGCCCTT 3′ and 5′ TCATAATCAGGAGAGAGGAGT 3′. The PCR-amplified fragments of *STAT4* that were 338 bp in length were isolated and digested with the endonuclease HpyCH4III (ACN/GT) NewEngland BioLabs, (Ipswich, USA). The *STAT4* C allele was cleaved into 258 and 80 bp fragments, whereas the *STAT4* G allele remained uncut. DNA fragments were separated by electrophoresis on 3 % agarose gel and visualized by ethidium bromide staining. The *STAT4* C > G polymorphism was confirmed by repeated PCR–RFLP. The genotyping quality was examined by direct sequencing of approximately 10 % of the all samples.

### Statistical analysis

The distribution of genotypes in patients and controls was examined for deviation from Hardy–Weinberg equilibrium using exact and log likelihood ratio χ^2^ tests (http://ihg.gsf.de/cgi-bin/hw/hwa1.pl). The polymorphism was tested for association with SLE incidence using the χ^2^ test for trend (*p*_trend_). The χ^2^ test was employed to examine differences in genotypic and allelic distribution between patients and controls, and a *p* value <0.05 was considered statistically significant. The Odds Ratio (OR) and 95 % Confidence Intervals (95 % CI) were calculated. Contribution of the *STAT4* C > G polymorphism to clinical manifestations and the production of autoantibodies (Ab) was determined by χ^2^ test. The Bonferroni correction for multiple comparisons was used and both *p* values, before (*p*) and after correction (*p*_corr_), were determined. Power analysis was performed using uncorrected χ^2^ test using Power and Sample Size Calculation program version 2.1.30.

## Results

### Prevalence of *STAT4 G* > *C* polymorphism in SLE patients and healthy individuals

Distribution of *STAT4* G > C genotypes did not display significant deviation from Hardy–Weinberg equilibrium between patients and healthy individuals. The prevalence of the *STAT4* C/C genotype was 1.8-fold times higher in patients with SLE than in healthy individuals (Table 2). The *STAT4* C/G heterozygous frequency in patients was higher than in controls and amounted to 37 and 31 %, respectively (Table 2). The OR for SLE patients with the C/C genotype as compared to the C/G and G/G genotypes was 1.967 (95 % CI = 1.152–3.358, *p* = 0.0119) and OR for the C/C and C/G genotypes as compared to the G/G genotype was 1.583 (95 % CI = 1.168–2.145, *p* = 0.0030) (Table 2; Figure 1S, online supplementary data).

**Table 2:** Prevalence of the *STAT4* G > C (rs7582694) polymorphisms in SLE patients and controls

*STAT4* G > C (rs7582694)	SLE n = 253 (%)	Controls n = 521 (%)	OR	95 %CI	*P* value^d^	*P*_trend_	Power
Genotype frequency							
G/G	131 (0.52)	328 (0.63)				0.0008	
C/G	94 (0.37)	162 (0.31)					
C/C	28 (0.11)	31 (0.06)	1.967^a^	1.152–3.358^a^	0.0119^a^		69
C/G + C/C	122 (0.48)	193 (0.37)	1.583^b^	1.168–2.145^b^	0.0030^b^		84
Minor allele frequency							
C	0.30	0.22	1.539^c^	1.209–1.959^c^	0.0004^c^		93

The Odds ratio was calculated for patients ^a ^(C/C vs C/G or G/G genotype), ^b^ (C/C or C/G vs G/G genotype). We also determined the OR for the patients’ minor allele; ^c^ (C allele vs G allele); ^d^ χ^2^ test

To evaluate the effect of the minor allele as a risk factor in SLE incidence, we also assessed the minor allele’s distribution in patients and healthy individuals. The frequency of the *STAT4* C allele was higher in patients with SLE compared to healthy individuals, with frequencies of 30 and 22 %, respectively (Table 2). The OR for the *STAT4* C allele frequency showed a 1.539-fold increased risk of SLE (95 % CI = 1.209–1.959, *p* = 0.0004) (Table 2; Figure 1S, online supplementary data). The p value of the χ^2^ test for the trend observed for the *STAT4 G* > *C* polymorphism was also statistically significant (*p*_trend_ = 0.0008). The statistical power of this study amounted to 84 % for the C/C or C/G genotypes and 69 % for the C/C genotype (Table 2).

### Contribution of *STAT4 G* > *C* polymorphism to clinical manifestations and production of autoantibodies in patients with SLE

We found an association between *STAT4* C/C and C/G genotypes with renal OR = 2.259 (1.365–3.738, *p* = 0.0014), (*p*_corr_ = 0.0238) and neurologic manifestations OR = 2.867 (1.467–5.604, *p* = 0.0016), (*p*_corr_ = 0.0272) of the disease (Table 1; Figure 2S, online supplementary data). Moreover, we observed a significant association between the *STAT4* C/C and C/G genotypes and the presence of anti-snRNP Ab OR = 3.237 (1.667–6.288, *p* = 0.0003), (*p*_corr_ = 0.0051). There was also significant association between the C/C and C/G genotypes and the anti-Scl-70 Ab OR = 2.665 (1.380–5.147, *p* = 0.0028), (*p*_corr_ = 0.0476) (Table 3; Figure 3S, online supplementary data).

**Table 3:** Contribution of the *STAT4* G > C *(rs7582694)* polymorphism to the presence of various autoantibodies in patients with SLE

Autoantibodies (aAb)	Genotype distribution			Odds ratio (95 % CI), *p* ^c^
	G/G	G/C	C/C	
	(131)^a^	(94)^a^	(28)^a^	
Anti-dsDNA	54	35	11	
Anti-Smith	12	8	3	
Anti-snRNP	15	31	5	3.237(1.667–6.288, *p* = 0.0003)^b^
Anti-Ro	21	17	5	
Anti-La	18	14	4	
Anti-Scl-70	16	28	5	2.665 (1.380–5.147, *p* = 0.0028)^b^

^a^Absolute number of positive patients for G/G, G/C, C/C. Genotype comparison ^b^(C**/**C or G**/**C vs G**/**G genotype) between patients with and patients without an autoantibody was performed by ^c^χ^2^ test. The autoantibody titers were determined by ELISA kit (EUROIMMUN AG, Germany) and were in the range from 100 to 700 IU/ml for anti-dsDNA, and in the range from 20 to 180 RU/ml for anti-Smith, anti-snRNP, anti-Ro, anti-La, and anti-Scl-70. The cut-off normal range was <100 IU/ml for anti-dsDNA and <20 RU/ml for other autoantibodies

## Discussion

STATs include DNA-interacting transcription factors that trigger the expression of the DNA’s target genes by recognizing specific DNA regulatory sequences [10]. The expression of *STATs* has been observed in a vast range of cell types, however the expression of STAT4 mainly takes place in immune cells and the testis [22]. STAT4 is essential for signal transduction by interleukin-12 (IL-12), interleukin-23 (IL-23), and type 1 interferon (IFN**)** in T cells and monocytes [10]. IL-12 induces the STAT4-dependent NK cell activation and differentiation of naive CD4^+^ lymphocytes into Th1 effector cells and IFNγ production [23–25]. STAT4 also mediates the IL-23-dependent expansion of Th17 cells, contributing to autoimmune diseases [26]. It has been demonstrated that STAT4-deficient mice display reduced manifestation of T cell-linked experimental autoimmune diseases including encephalomyelitis, arthritis, myocarditis, colitis, and autoimmune diabetes [10]. Moreover, *STAT4* deficiency results in a reduction of IFNγ biosynthesis in immune cells [10]. Accordingly, an association between disease activity in SLE patients and activation of the type 1 IFN system has been observed [27].

We observed that *STAT4 G* > *C* (rs7582694) intronic substitution may significantly increase the risk of SLE occurrence in a sample of the Polish population. Recent studies carried out by Luan et al. [28] demonstrated a statistically significant contribution of *STAT4 G* > *C* (rs7582694) to SLE incidence in the Mainland Chinese female population. The association of the *STAT4* *G* > T (rs7574865) polymorphism with SLE development was also previously observed in other Asian ethnic groups residing in Hong Kong, Northern Han of China, and Japan [14–19]. The contribution of the *STAT4 G* > *C* (rs7582694) or *STAT4* *G* > T (rs7574865) polymorphisms to SLE incidence was also observed in large groups of patients of European origin, among them a Finnish family cohort as well as Spanish, Swedish and other populations [11–13, 29, 30]. The SNP rs7574865 has also been confirmed as a genetic risk factor in the incidence of SLE in Colombian, Mexican, and Argentinian cohorts [30, 31]. However, the rs7574865 polymorphism has not been found to be a risk factor for SLE in the Turkish population [32].

In our study, patients with the *STAT4* C/C or G/C genotypes exhibited a significantly increased risk of developing either renal or neurologic manifestations of SLE.

An association between the *STAT4 G* > *T* (rs7574865) polymorphism and nepthritis has also been demonstrated in Americans of European descent [11]. Moreover, the contribution of *STAT4* G > C (rs7582694) or *STAT4* G > T (rs7574865) SNPs to the production of double-stranded DNA autoantibodies has been found in Swedish and American Europeans [11, 12, 33]. The *STAT4* G > T (rs7574865) SNP has also been associated with antiphospholipid syndrome in Italian cohorts [34], as has the production of anti-Sm antibodies in a Northern Han Chinese population [15]. Additionally, other STAT4 SNPs were correlated with lupus nephritis, arthritis, and the production of anti-SSA/B autoantibodies in a Northern Han Chinese population [15]. The different effects of the *STAT4* G > C (rs7582694) or *STAT4* G > T (rs7574865) SNPs on clinical manifestations in various ethnicities may result from different sizes of the studied groups, genetic heterogeneity or patient interaction with disparate environmental factors [6].

*STAT4* gene variants have also been found to be risk factors for other autoimmune diseases including rheumatoid arthritis, Crohn’s disease, asthma, systemic sclerosis, and Sjogren’s syndrome [35–39].

The role of *STAT4 G* > *C* (rs7582694) or *STAT4* *G* > T (rs7574865) SNPs in the expression of *STAT4* have been studied [12, 30]. Abelson et al. (2009) used quantitative PCR to demonstrate significantly higher levels of STAT4 mRNA in mononuclear cells bearing the SLE risk TT genotypes as compared to cells having the GG or GT genotypes of the rs7574865 SNP [30]. Moreover, Sigurdsson et al. [12] indicated that gene variants having the rs8179673 SNP in almost perfect pair-wise LD with rs7582694 led to over-expression of *STAT4* in pooled primary human osteoblasts.

Our genetic studies are consistent with other studies that have demonstrated the *STAT4 G* > *C* (rs7582694) intronic substitution as a significant risk factor of SLE incidence. Moreover, we found that this SNP can be associated with renal and neurological symptoms of SLE. Since this autoimmune disease is vastly heterogeneous, further studies of this polymorphism’s effects on clinical manifestations of SLE in other populations would be valuable.

## Electronic supplementary material

Below is the link to the electronic supplementary material.

Supplementary material1 (JPG 90 mb)

Supplementary material 2 (JPG 3.12 mb)

Supplementary material 3 (JPG 2.22 mb)

Supplementary material 4 (DOCX 15 kb)
