# Supplementary figures and images for: Contribution of STAT4 gene single-nucleotide polymorphism to systemic lupus erythematosus in the Polish population
Source: Mol Biol Rep. 2012 Jun 24;39(9):8861–6. doi: 10.1007/s11033-012-1752-3 (PMC3404285; doi:10.1007/s11033-012-1752-3)

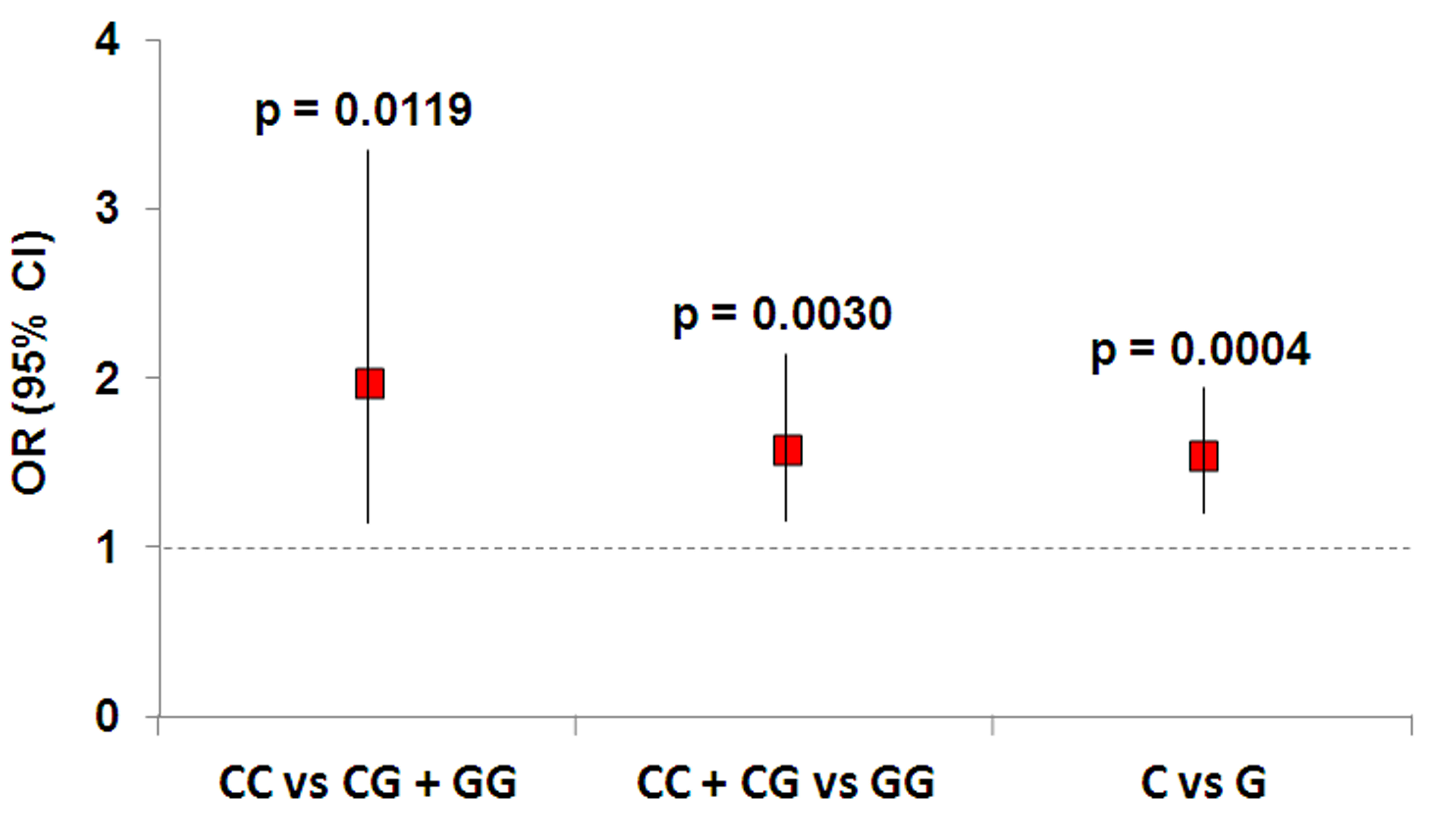

Supplement: Supplementary file 1 — Supplementary material1 (JPG 90 mb) [file 11033_2012_1752_MOESM1_ESM.jpg]

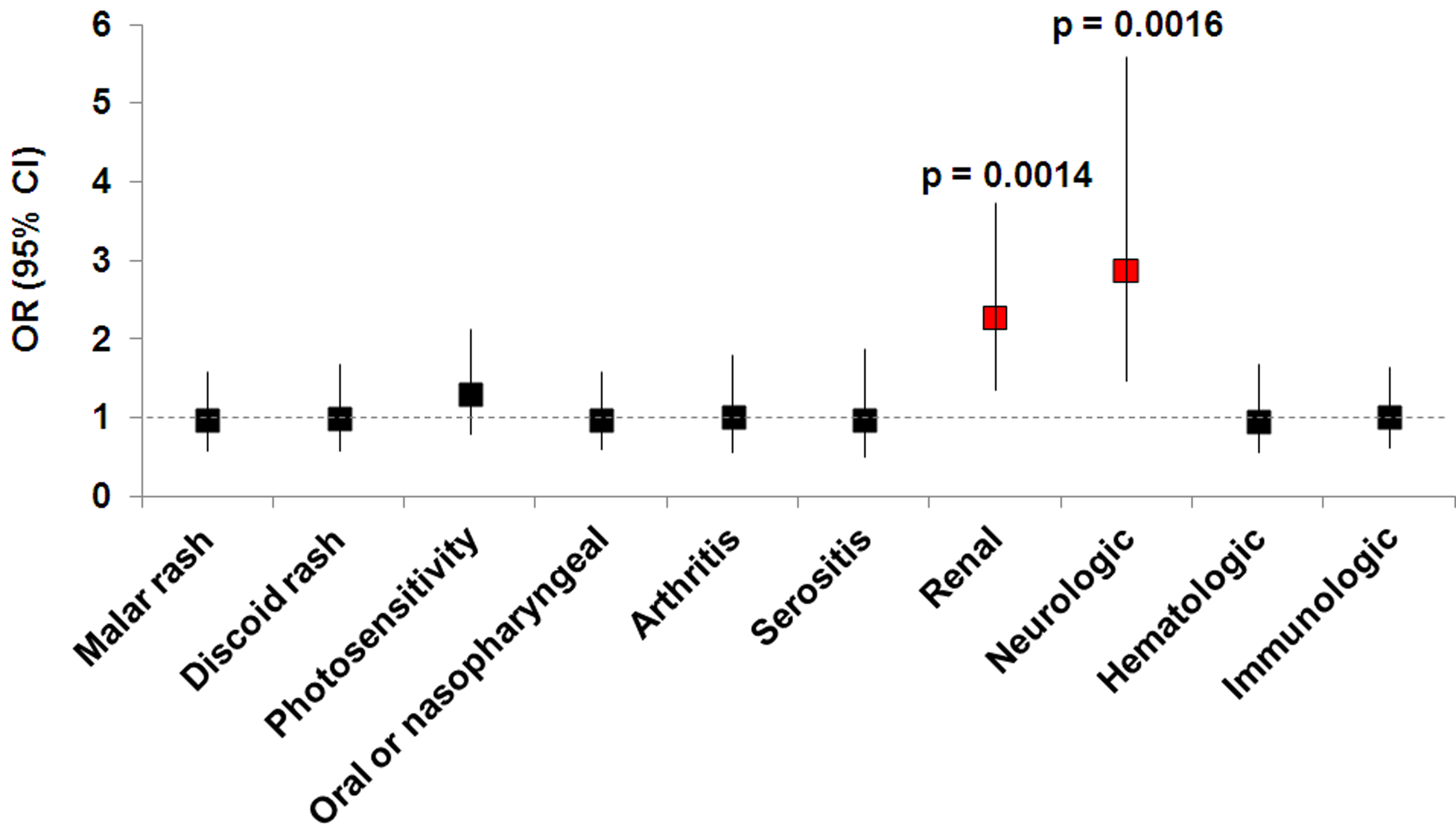

Supplement: Supplementary file 2 — Supplementary material 2 (JPG 3.12 mb) [file 11033_2012_1752_MOESM2_ESM.jpg]

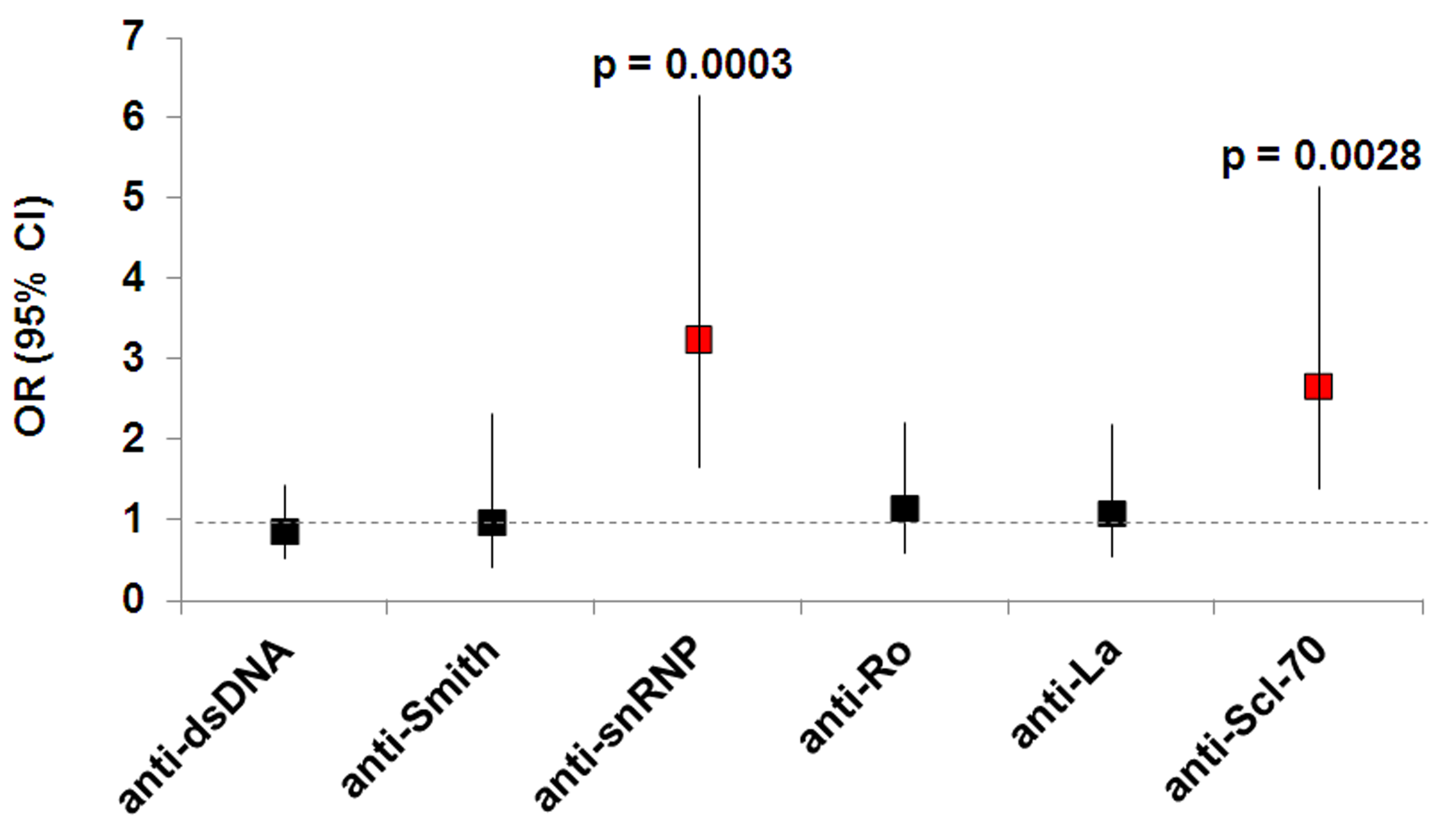

Supplement: Supplementary file 3 — Supplementary material 3 (JPG 2.22 mb) [file 11033_2012_1752_MOESM3_ESM.jpg]
