# Supplementary material for: Contribution of STAT4 gene single-nucleotide polymorphism to systemic lupus erythematosus in the Polish population
Source: Mol Biol Rep. 2012 Jun 24;39(9):8861–6. doi: 10.1007/s11033-012-1752-3 (PMC3404285; doi:10.1007/s11033-012-1752-3)
Supplement: Supplementary file 4 — Supplementary material 4 (DOCX 15 kb) [file 11033_2012_1752_MOESM4_ESM.docx]

**Figure legends**

**Figure 1S, online supplementary data**

Odds Ratio (OR) plot for genotyping and allelic frequencies of the *STAT4* G/C polymorphism in patients and controls. Each OR value is represented by the corresponding square with arms representing 95% Confidence Intervals (95% CI). The analysis was performed by χ^2^ test. The red box indicates a significant association.

**Figure 2S, online supplementary data**

Odds Ratio (OR) plot for comparison of genotypes *STAT4* C**/**C or G**/**C vs G**/**G between patients with and patients without a particular manifestation. Each OR value is represented by the corresponding black or red square with arms representing 95% Confidence Intervals (95% CI). The analysis was performed by χ^2^ test. The red box indicates a significant association.

**Figure 3S, online supplementary data**

Odds Ratio (OR) plot for comparison of genotypes *STAT4* C/C or G/C vs G/G between patients with and patients without an autoantibody. Each OR value is represented by the corresponding black or red square with arms representing 95% Confidence Intervals (95% CI). The analysis was performed by χ^2^ test. The red box indicates a significant association.
